# Failure to integrate: Connector hub dysfunction in major depressive disorder

**DOI:** 10.1016/j.nicl.2026.104010

**Published:** 2026-05-19

**Authors:** Norika Hayashi, Epifanio Bagarinao

**Affiliations:** a Department of Nursing, School of Health Sciences, Faculty of Medicine, Nagoya University, Nagoya, Aichi, Japan; b Brain and Mind Research Center, Nagoya University, Nagoya, Aichi, Japan; c Department of Integrated Health Sciences, Nagoya University Graduate School of Medicine, Nagoya, Aichi, Japan

**Keywords:** Connector hubs, Major depressive disorder, Resting-state fMRI, Functional connectivity overlap ratio, Resting-state networks, Functional connectivity

## Abstract

Major depressive disorder (MDD) is a highly prevalent and disabling psychiatric condition characterized by varying degrees of affective, cognitive, somatic, and psychomotor symptoms. Neuroimaging studies have identified dysfunctional connections of several large-scale brain networks in MDD that have been associated with its various symptoms. Given the involvement of multiple brain networks, we hypothesized that MDD could be driven by connectivity impairments of key regions involved in the brain’s integrative processes called connector hubs. MRI data from 255 patients with MDD and 255 matched healthy controls were used in the analysis. A network metric called functional connectivity overlap ratio, computed from resting-state functional MRI, was used to identify affected hub regions. Our results showed that connector hubs located in the sensorimotor cortex, thalamus, cerebellum, and limbic regions exhibited altered connections across multiple networks in patients compared to controls. Specifically, sensorimotor connector hubs had significantly less connections with primary processing (sensorimotor, visual, and auditory), default mode, and visuospatial networks, but more widespread connections with the anterior salience and basal ganglia networks. Cerebellar and thalamic connector hubs exhibited less connections with the core neurocognitive (default mode, executive control, and salience), language, and basal ganglia networks, whereas limbic connector hubs had impaired connections with salience, language, and basal ganglia networks. Additionally, some of these altered connections correlated with depression scores in patients. Together, these findings suggest that MDD is characterized by a more widespread impairment of the brain’s integrative functions involving sensory, motor, cognitive, and affective processes, reflecting its diverse symptoms.

## Highlights

• MDD is characterized by widespread, multi-network connector hub disruptions.
• Impaired hubs found in sensorimotor cortex, thalamus, cerebellum, and limbic areas.
• Basal ganglia network showed the most widespread connectivity alterations.
• Some altered connections correlated with depression severity scores.

## 1 Introduction

Major depressive disorder (MDD) is a highly prevalent and disabling psychiatric condition characterized by varying degrees of affective (Association, 2022, Insel et al., 2010), cognitive (Trivedi and Greer, 2014), somatic (Buysse et al., 2008, Heinzel et al., 2009, Tsuno et al., 2005), and psychomotor (Schrijvers et al., 2008, Sobin and Sackeim, 1997) symptoms, making it a highly heterogeneous disorder. To understand its neuropathological background, several studies have investigated structural and functional alterations in the brain associated with MDD using neuroimaging data. Specifically, studies examining brain connectivity alterations in MDD patients have identified dysfunctional connections within and between different large-scale functional networks (Kaiser et al., 2015, Yan et al., 2019). Thus, the pathophysiology of MDD is increasingly understood in terms of dysregulated communication among distributed brain networks, rather than isolated regional dysfunction.

Central to this view is the triple network model, which implicates the default mode network (Berman et al., 2011, Hamilton et al., 2015, Sheline et al., 2009), the executive control network (Fales et al., 2008), and the salience network (Javaheripour et al., 2021, Kaiser et al., 2015, Manoliu et al., 2014). These networks’ putative functions aligned well with the different symptoms observed in MDD including rumination associated with the observed hyperconnectivity within the default mode network (Hamani et al., 2011, Kaiser et al., 2015, Sheline et al., 2010), emotional disinhibition with the hypoconnectivity of the executive control network with other networks (Kaiser et al., 2015), and emotional over-reactivity with the hyperconnectivity of the salience network with affective networks (Berman et al., 2011, Hamilton et al., 2015, Kaiser et al., 2015).

Aside from the involvement of the core neurocognitive networks (default mode, executive control, and salience networks), recent studies have also implicated networks associated with primary processing, such as sensorimotor and visual networks, in MDD (Javaheripour et al., 2021, Ray et al., 2021, Yan et al., 2019). In a mega-analysis using resting-state functional MRI data, Javaheripour, et al. reported significant hypoconnectivity within the sensorimotor network (Javaheripour et al., 2021). Additionally, this network was also shown to have significantly lower network segregation in MDD patients. Altered effective connectivity was also found in sensorimotor cortices and shown to be robust across different datasets (Ray et al., 2021).

Given the observed connectivity alterations involving several large-scale functional networks in MDD, a framework to unify these various changes is needed. In this study, we hypothesized that MDD could be driven by dysfunction of key regions involved in the brain’s integrative processes called connector hubs. These regions have widespread connections across multiple large-scale functional networks, are pivotal in the coordination of information flow (Sporns et al., 2007) among networks, and are crucial for the integration of functionally specialized systems. Studies have shown that disruption of these regions by lesion (Gratton et al., 2012) or by non-invasive transcranial magnetic stimulation (Lynch et al., 2019) could impact brain network function. As such, their impairments have been associated with neurological and psychiatric disorders (Bagarinao et al., 2024, Bagarinao et al., 2022, van den Heuvel and Sporns, 2013, Yamamoto et al., 2022). The identification of affected connector hub regions is therefore critical in understanding failures in global network integration and domain-specific deficits observed across motor, cognitive, and affective domains in MDD. Additionally, identified connector hubs can also serve as potential regions that could be targeted for treatment to normalize brain network functions.

To test our hypothesis, we extensively examined connectivity alterations across the whole brain using functional connectivity overlap ratio (FCOR) (Bagarinao et al., 2020), a network metric that can be estimated from resting-state functional magnetic resonance imaging (MRI) data. The FCOR technique expands traditional seed-based or network-wise analyses by quantifying the spatial extent of a region’s connections with the different large-scale functional networks (region-to-network connectivity), making it particularly sensitive to connector hub identification. Additionally, it can also capture both the breadth and the specificity of altered hub-level integration in terms of which large-scale networks are affected. Moreover, it can be used to identify connector hub regions at the voxel-level resolution across the whole brain (Bagarinao et al., 2020, Kawabata et al., 2022, Kawabata et al., 2021) including subcortical and cerebellar areas. Using resting-state functional MRI data, whole-brain FCOR maps for several established resting-state networks (RSNs) were generated and used to examine alterations in region-to-network connectivity in the patient group. Affected connector hubs were then identified by examining regions with significant FCOR alterations across multiple networks and validated using seed-based connectivity analyses.

## 2 Methods and materials

### 2.1 Participants

We used MRI data from the Strategic Research Program for the Promotion of Brain Sciences (SRPBS) Multi-Disorder MRI Dataset (unrestricted, https://bicr-resource.atr.jp/srpbsopen/) (Tanaka et al., 2021). The dataset consisted of structural (T1-weighted) MRI, resting-state functional MRI, demographic information such as age and sex, and clinical rating scales of 1410 participants collected from eight imaging sites in Japan. Of the 1410 participants, 791 were healthy controls and 619 were patients across seven disorders. From this pool of participants, patients with MDD (N = 255) and their corresponding age- and sex-matched healthy controls (N = 255), identified using propensity score matching (Austin, 2011) as implemented in *R*, were extracted. The characteristics of the selected participants are summarized in Table 1. All participants provided written informed consent at their local institutions, and all experimental protocols were approved by the principal investigators’ respective institutional review boards.

**Table 1:** Participants’ characteristics.

Site	Group	N (Male/Female)	Age (years) Mean (SD)	Mean FD (mm) Mean (SD)	BDI-II Mean (SD)
All	HC	255 (126/129)	42.38 (13.94)	0.18 (0.15)	7.56 (6.52)
	MDD	255 (135/120)	42.64 (12.16)	0.17 (0.12)	27.61 (10.40)
	p-value	−	0.54	0.11	**2.41 × 10^-54^**
HUH	HC	59 (29/30)	33.41 (12.90)	0.12 (0.12)	6.71 (5.53)
	MDD	57 (32/25)	43.33 (12.18)	0.10 (0.05)	30.89 (9.04)
	p-value	−	**8.90 × 10^-6^**	0.24	**7.89 × 10^-20^**
HRC	HC	17 (7/10)	36.53 (9.77)	0.11 (0.04)	11.41 (9.98)
	MDD	16 (6/10)	40.50 (11.48)	0.11 (0.04)	35.31 (9.52)
	p-value	−	0.35	0.79	**1.64 × 10^-5^**
HKH	HC	29 (12/17)	45.41 (9.53)	0.15 (0.09)	5.14 (4.58)
	MDD	33 (20/13)	44.82 (11.47)	0.15 (0.09)	28.52 (8.67)
	p-value	−	0.72	0.94	**5.13 × 10^-11^**
COI	HC	74 (35/39)	48.15 (12.91)	0.29 (0.19)	7.93 (6.39)
	MDD	71 (31/40)	45.17 (12.46)	0.27 (0.14)	26.14 (9.89)
	p-value	−	0.12	0.44	**8.57 × 10^-20^**
KUT	HC	16 (10/6)	42.81 (12.77)	0.20 (0.09)	−
	MDD	16 (10/6)	42.56 (12.48)	0.14 (0.06)	27.31 (10.32)
	p-value	−	0.95	0.07	−
UTO	HC	60 (33/27)	44.18 (14.71)	0.15 (0.08)	11.12 (7.49)
	MDD	62 (36/26)	38.74 (11.62)	0.15 (0.12)	20.38 (11.41)
	p-value	−	0.12	0.27	**0.041**

Abbreviations: COI, Center of Innovation, Hiroshima University (VerioDot, Siemens); HKH, Hiroshima Kajikawa Hospital (Spectra, Siemens); HRC, Hiroshima Rehabilitation Center (Signa HDxt, GE); HUH, Hiroshima University Hospital (Signa HDxt, GE); KUT, Kyoto University (TimTrio, Siemens); UTO, University of Tokyo (MR750W, GE); BDI-II, Beck Depression Inventory – II; FD, frame-wise displacement; HC, healthy controls; MDD, patients with major depressive disorder; SD – standard deviation.

NOTES: 1. p-values were computed using Wilcoxon rank sum test for equal medians. 2. Not all participants have BDI-II scores. The number of participants included in the computation of BDI-II column is as follows: All (HC/MDD) = 186/225, HUH = 59/57, HRC = 17/16, HKH = 29/33, COI = 73/71, KUT = 0/16, and UTO = 8/32.

### 2.2 Magnetic resonance imaging data and preprocessing

The T1-weighted MRI and resting-state functional MRI data were extracted from the SRPBS data repository. The details of the imaging parameters from the different sites are given in the Supplementary Materials (Imaging Parameters). All images were preprocessed using Statistical Parametric Mapping (SPM12, Wellcome Trust Center for Neuroimaging, London, UK) software running on Matlab (R2021b, MathWorks, Natick, Mass, USA) and following the preprocessing pipeline used in our previous paper (Bagarinao et al., 2020). Briefly, each T1-weighted image was segmented to extract the transformation information necessary to normalize images from the individual subject space to the Montreal Neuroimaging Institute (MNI) template space. Using this information, the resting-state functional MRI data was normalized to the MNI space after slice-timing correction, realignment, and co-registration. Normalized images were additionally smoothed, resampled to 3 × 3 × 3 mm^3^ resolution, corrected for the effects of head motion and other physiological noise, and bandpass filtered within 0.01 – 0.1 Hz.

### 2.3 Functional connectivity overlap ratio

Using the preprocessed resting-state functional MRI data, individual FCOR maps (Bagarinao et al., 2020) were generated. FCOR quantifies the spatial extent of a voxel’s connection to a given RSN template and can range from 0, for no connection, to 1, when the voxel is fully connected to all the nodes in the template. In this study, we used 14 RSN templates (Shirer et al., 2012) consisting of the core neurocognitive networks including the dorsal default mode network (dDMN), ventral default mode network (vDMN), precuneus network (Prec), anterior salience network (aSal), posterior salience network (pSal), left executive control network (LECN), and right executive control network (RECN); the primary processing networks including sensorimotor network (SMN), primary visual network (pVis), high visual network (hVis), and auditory network (Aud); and other networks including visuospatial network (Visu, dorsal attention network), language network (Lang) and basal ganglia network (BG). This set of RSNs was selected since it has whole-brain coverage including subcortical areas and cerebellum, is based on volume data, and extensively covers all known RSNs. For each RSN, FCOR maps were generated for all participants. FCOR values across the whole brain were then converted into z-scores for statistical comparisons (Bagarinao et al., 2020, Buckner et al., 2009). To account for potential variations in FCOR values due to differences in imaging parameters and scanners used across multiple sites, the constructed FCOR maps were further harmonized using ComBat (Fortin et al., 2018, Fortin et al., 2017) before statistical analysis.

### 2.4 Statistical analysis

#### 2.4.1 Whole-brain FCOR analysis

To identify changes in FCOR values across the whole brain, for each RSN, a two-sample *t*-test was performed between patient and control groups using the RSN’s harmonized FCOR maps. We included age, sex, and mean frame-wise displacement (Power et al., 2014), representing head motion during resting-state functional MRI scans, as covariates of no interest. The statistical significance of the resulting contrast maps (control > MDD and control < MDD) was assessed using p < 0.05 corrected for multiple comparisons using family-wise error correction at the cluster level (FWEc) with a cluster-defining threshold (CDT) set at p = 0.001 as implemented in SPM12. No additional corrections were made across RSNs. BrainNet viewer (Xia et al., 2013) was used to generate images for contrast maps.

#### 2.4.2 Conjunction analysis

To identify potential connector hub regions affected by MDD, we performed conjunction analysis across the different RSNs using the contrast maps from whole-brain FCOR analysis. Specifically, the obtained contrast maps were binarized by assigning a value of 1 to voxels with significant FCOR alterations between patients and controls and 0 to others. The binarized contrast maps from the 14 RSNs were then combined using voxel-wise summation, such that in the combined map, the value of each voxel represents the number of RSNs where significant difference in FCOR values between patients and controls was observed. From the conjunction map, peaks with values ≥ 3 were identified and voxels with the peak value were extracted and defined as a region-of-interest (ROI) for the succeeding analysis. These ROIs were used to represent the affected connector hub regions. The maps representing the contrasts control > MDD and control < MDD were separately analyzed.

#### 2.4.3 Region-of-interest analysis

To examine the connectivity of affected connector hubs, the ROIs identified from the conjunction analysis were used for further analysis. For each ROI, seed-based connectivity analysis was performed to construct the ROI’s functional connectivity map. The constructed connectivity maps were also harmonized using ComBat to account for site differences. Using the harmonized maps, two sample t-tests were performed to identify differences in the connectivity of the ROI across the whole brain between patients and controls. For this analysis, we also included age, sex, and the mean frame-wise displacement as covariates of no interest. Significance was assessed using FWEc p < 0.05 with a CDT set at p = 0.001 at the ROI level. No additional corrections were made across ROIs.

Furthermore, the harmonized FCOR values associated with the 14 RSNs were also extracted from all voxels in each ROI and the mean value was computed. Each ROI was therefore represented by 14 mean FCOR values, one for each RSN. To assess their clinical significance, we examined the relationship between the extracted mean FCOR values and the patients’ Beck Depression Inventory-II (BDI-II) scores using nonparametric Spearman’s correlation coefficient. Age, sex, and mean frame-wise displacement were regressed from FCOR values before correlation analysis. Significance was assessed using uncorrected p < 0.05.

To identify the networks where the identified connector hub ROIs have significant FCOR alterations, we also systematically re-evaluated the difference of the mean FCOR values for each RSN between patients and controls (ROI level comparisons). Specifically, for each ROI, the mean FCOR values for each RSN were compared between patients and controls using the nonparametric Wilcoxson rank sum test. Age, sex, and mean frame-wise displacement were first regressed from FCOR values before the comparison. Significance was assessed using false discovery rate correction at q < 0.05, controlling for multiple comparisons across ROIs and RSNs.

## 3 Results

### 3.1 Participants’ characteristics

Across all sites, no significant difference was observed in age between patients with MDD and controls. However, MDD patients from HUH were significantly (p = 8.90 × 10^−6^, Wilcoxon rank sum test) older than controls from the same site. In terms of head motion, the average mean frame-wise displacement was less than or equal to 0.2 mm for all sites except COI. There was also no significant difference in mean frame-wise displacement between groups. BDI-II scores differed significantly (p = 2.41 × 10^−54^, Wilcoxon rank sum test) between patients and controls across all sites. The participants’ characteristics are summarized in Table 1. Histograms for age, sex, and mean frame-wise displacement values are given in Supplementary Fig. S1.

### 3.2 Whole-brain FCOR alterations

**Fig. 1:**
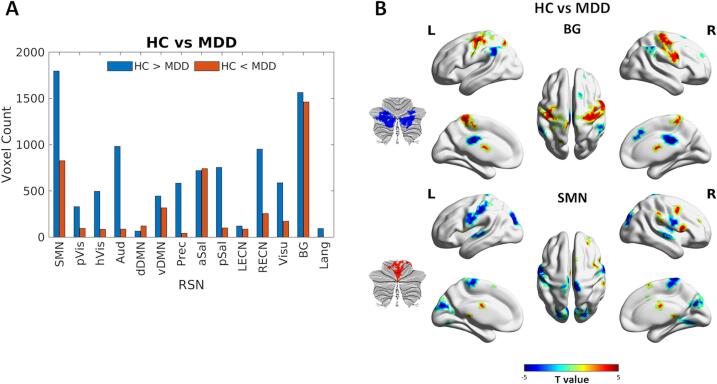
Whole-brain region-to-network connectivity alterations in patients with MDD compared to healthy controls. A) Number of voxels (voxel count) showing significant alterations in FCOR values with several resting-state networks (RSN) in patients with MDD compared to healthy controls (HC). Blue bars indicate the number of voxels with lower FCOR values, whereas red bars indicate voxels with higher FCOR values in the patient group. B) Contrast maps showing actual locations of regions exhibiting significant alterations in FCOR values with BG (top figure) and SMN (lower figure). Regions with lower FCOR values in patients (HC > MDD) are shown in blue, whereas those with higher FCOR values (HC < MDD) are shown in red. The contrast maps of the remaining RSNs are shown in Supplementary Fig. S2. The list of clusters including the peak’s MNI coordinate and cluster size is given in Supplementary Table S1. (For interpretation of the references to colour in this figure legend, the reader is referred to the web version of this article.)

Fig. 1A shows the number of voxels with altered connections to each RSN in the patient group compared to the control group. Among the networks examined, SMN has the highest number of voxels showing significantly lower FCOR values in patients, followed by BG, then Aud, RECN, salience (pSal, aSal) and others. By contrast, BG has the highest number of voxels with significantly higher FCOR values in patients compared to controls, followed by SMN, and aSal. The actual locations of regions with altered FCOR values are shown in Fig. 1B for BG and SMN. Specifically, regions showing lower FCOR values with the BG in patients included the bilateral cerebellum, bilateral supramarginal gyrus, bilateral midcingulate gyrus, bilateral thalamus, right middle frontal gyrus, and right superior frontal gyrus. By contrast, regions showing higher FCOR values included the bilateral precentral gyrus, left superior parietal lobule, and left thalamus. For SMN, lower FCOR values were observed in sensorimotor (postcentral gyrus, precentral gyrus, paracentral lobule), occipital (cuneus, superior, lingual, occipital fusiform gyri), and temporal (superior temporal gyrus, temporal pole) regions in the patient group. On the other hand, higher FCOR values were observed in the right opercular part of the inferior frontal gyrus, cerebellum, midcingulate gyrus, and others. The list of all clusters showing significant alterations in FCOR values for all RSNs in patients with MDD compared to healthy controls is given in Supplementary Table S1. The contrast maps for the remaining RSNs are also shown in Supplementary Fig. S2.

### 3.3 Alterations in connector hub regions

**Fig. 2:**
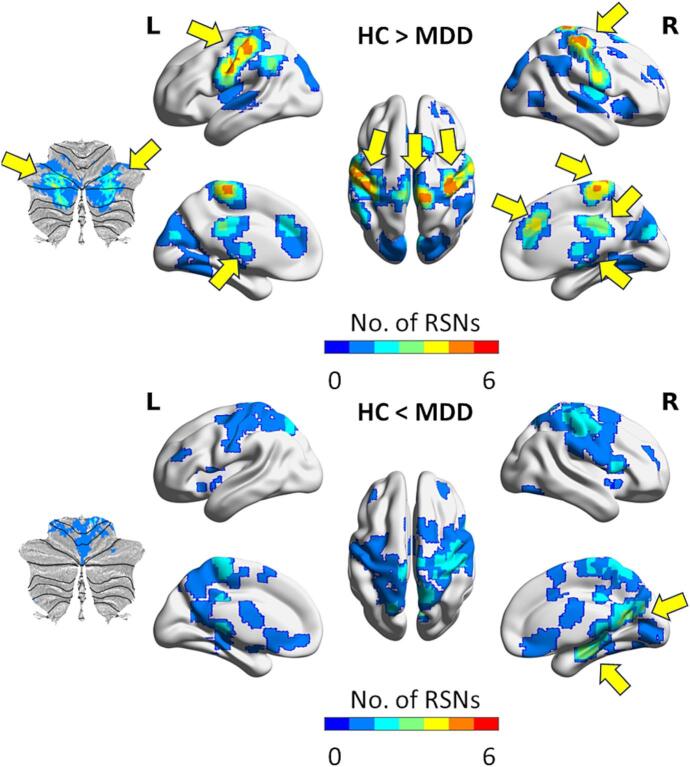
Conjunction maps of the two contrasts (HC > MDD and HC < MDD) summarizing all regions with altered connections to one or more resting state networks (RSNs) in MDD patients. Voxel values represent the number of RSNs where altered FCOR values were significant. Yellow arrows indicate regions with altered connections with 3 or more RSNs. Some of the regions (e.g., the ventral lateral nucleus of the thalamus) may not be displayed prominently in this surface projection. The list of these regions is given in Table 2. (For interpretation of the references to colour in this figure legend, the reader is referred to the web version of this article.)

Fig. 2 shows the conjunction maps summarizing the different regions with altered FCOR values across RSNs. From these maps, several connector hubs exhibiting altered connections across multiple RSNs can be identified (Table 2). Specifically, for patients with MDD, the bilateral ventral lateral nucleus (L/R VLN) of the thalamus, left postcentral gyrus (L PoG), right supplementary motor area (R SMA), bilateral precentral gyrus (L/R PrG), bilateral posterior cerebellum (L/R pCer), right dorsal anterior cingulate cortex (R dACC), right midcingulate gyrus (R MCgG), and right putamen (R Pu) exhibited significantly lower FCOR values with at least 3 RSNs, whereas the right parahippocampal gyrus (R PHG) and the right posterior cingulate cortex (R PCC) showed more widespread connections (higher FCOR values) with at least 3 RSNs.

**Table 2:** Identified connector hub regions with FCOR alterations in 3 or more resting-state networks.

ROI	Contrast	Xc	Yc	Zc	Size (voxels)	Affected RSNs
L PoG	HC > MDD	-46	–22	51	45	SMN, hVis, Aud, vDMN, aSal*^)^, Visu, BG*^)^
R SMA	HC > MDD	6	−31	67	38	SMN, pVis, hVis, Aud, vDMN, Prec, aSal*^)^, Visu
L PrG	HC > MDD	−57	−8	32	8	SMN, hVis, Aud, vDMN, aSal*^)^, Visu, BG*^)^
R PrG	HC > MDD	37	−24	57	14	SMN, pVis, hVis, Aud, vDMN, Prec, aSal*^)^, Visu
L VLN	HC > MDD	−14	−14	11	30	Prec, aSal, pSal, LECN, RECN, BG, Lang
R VLN	HC > MDD	14	−9	11	11	dDMN, Prec, aSal, pSal, LECN, RECN, BG, Lang
L pCer	HC > MDD	−30	−68	–32	12	dDMN, Prec, aSal, pSal, LECN, RECN, BG, Lang
R pCer	HC > MDD	28	−70	–33	11	vDMN, Prec, aSal, pSal, LECN, RECN, BG
R MCgG	HC > MDD	3	−31	32	14	aSal, pSal, BG
R Pu	HC > MDD	22	4	−4	4	aSal, pSal, RECN, Lang
R dACC	HC > MDD	5	33	31	22	aSal, pSal, BG, Lang
R PCC	HC < MDD	11	−59	16	10	dDMN*^)^, LECN*^)^, RECN*^)^, BG*^)^
R PHG	HC < MDD	26	−20	−17	13	SMN, dDMN*^)^, vDMN*^)^, LECN*^)^, RECN*^)^

Abbreviations: aSal, anterior salience network; Aud, auditory network; BG, basal ganglia network; dACC, dorsal anterior cingulate cortex; dDMN, dorsal default mode network; FCOR, functional connectivity overlap ratio; HC, healthy control; hVis, high visual network; L, left; Lang, language network; LECN, left executive control network; MCgG, midcingulate gyrus; MDD, patients with major depressive disorder; PCC, posterior cingulate cortex; pCer, posterior cerebellum; PHG, parahippocampal gyrus; PoG, postcentral gyrus; Prec, precuneus network; PrG, precentral gyrus; pSal, posterior salience network; Pu, putamen; pVis, primary visual network; R, right; RECN, right executive control network; ROI, region-of-interest; RSNs, resting-state networks; SMA, supplementary motor area; SMN, sensorimotor network; vDMN, ventral default mode network; Visu, visuospatial network; VLN, ventral lateral nucleus;

NOTES: *) indicates higher FCOR values in patients compared to controls. Xc, Yc, Zc represents the MNI coordinates of the region’s center of gravity, rounded to the nearest integer value.

**Fig. 3:**
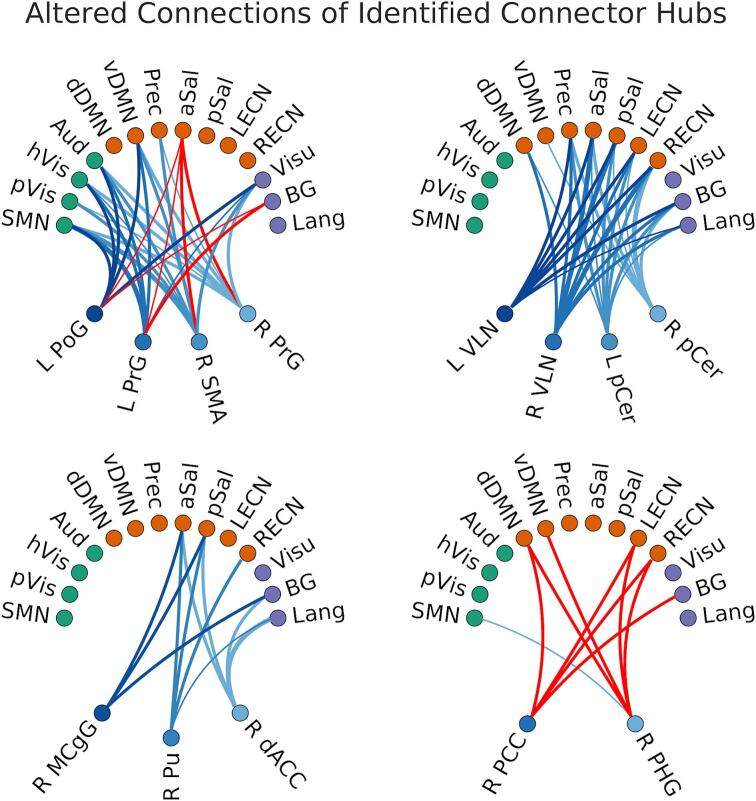
Altered connections between identified connector hub regions and the different resting-state networks. Blue lines indicate lower FCOR values in patients compared to controls, whereas red lines indicate higher FCOR values. The list of these regions is given in Table 2. (For interpretation of the references to colour in this figure legend, the reader is referred to the web version of this article.)

The affected connections of these connector hub regions are summarized in Fig. 3 and Table 2. Sensorimotor connector hubs (L PoG, R SMA, and L/R PrG; Fig. 3 top left) have lower FCOR values with primary processing (SMN, pVis, hVis, and Aud), default mode (vDMN, Prec), and Visu networks but higher FCOR values with aSal and BG networks. Thalamic and cerebellar connector hubs (L/R VLN and L/R pCer; Fig. 3 top right) had lower FCOR values primarily with the core neurocognitive networks (default mode, salience, and executive control) as well as with BG and Lang networks. The connector hub located in midcingulate gyrus had lower FCOR values with salience (aSal, pSal) and BG networks, that in the putamen with salience (aSal, pSal), RECN, and Lang networks, and that in the right dorsal anterior cingulate cortex with salience (aSal, pSal), BG, and Lang networks (Fig. 3 bottom left). The right PCC connector hub had higher FCOR values with the dDMN, executive control (RECN, LECN) and BG networks (Fig. 3 bottom right). Finally, the right parahippocampal gyrus connector hub had higher FCOR values with default mode (dDMN, vDMN) and executive control (LECN, RECN) networks, but lower FCOR values with SMN network.

### 3.4 Seed-based connectivity analysis

**Fig. 4:**
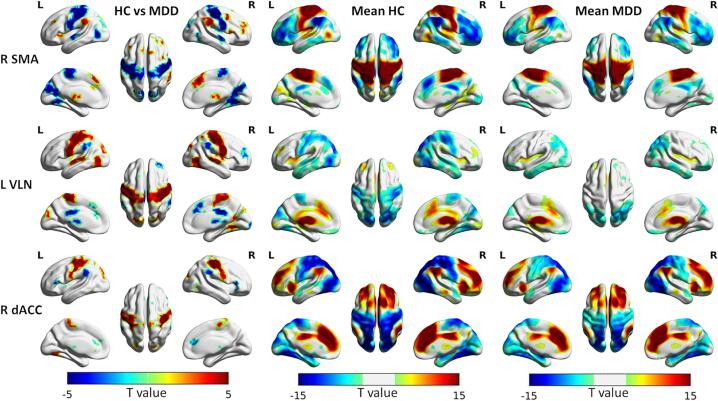
Seed-based connectivity analysis. Left column: contrast maps showing regions exhibiting significant difference in functional connectivity between patients with MDD and healthy controls (HC) for representative connector hubs located in the right supplementary motor area (R SMA, top row), left ventrolateral nucleus (L VLN) of the thalamus (middle row), and right dorsal anterior cingulate cortex (R dACC, bottom row). Blue colors indicate lower connectivity in the patient group, whereas red colors indicate higher connectivity. The mean functional connectivity maps for the control group are shown in the middle column (Mean HC), whereas those of the patient group are shown in the rightmost column (Mean MDD). For the mean connectivity maps, red colors indicate positive connectivity values (positive correlation), whereas blue colors indicate negative connectivity values (anti-correlation). (For interpretation of the references to colour in this figure legend, the reader is referred to the web version of this article.)

Changes in the functional connectivity of the identified connector hubs were examined using seed-based connectivity analysis. The results of the analysis are summarized in Fig. 4 for representative connector hubs. Sensorimotor connector hubs including R SMA exhibited significantly lower connectivity with primary processing systems (sensorimotor, visual, and auditory) in patients compared with controls (Fig. 4, top row). In addition, the observed anti-correlation of these hub regions with regions of the salience and executive control networks was also significantly lower in the patient group. On the other hand, the connectivity of thalamic and cerebellar connector hubs including L VLN was significantly lower with regions of the salience and default mode networks and their anti-correlation with regions associated with primary processing was also significantly lower in patients (Fig. 4, middle row). Similar decreases in the anti-correlation with primary processing networks were observed with the R dACC connector hub (Fig. 4, bottom row). Overall, in patients, the connectivity values were shifting towards lower values for both positive and negative correlations suggesting dysconnectivity in both directions. Functional connectivity and contrast maps for the rest of the connector hubs are shown in Supplementary Fig. S3.

### 3.5 Association of altered FCOR values with BDI-II scores in patients

Finally, we examined the association between altered FCOR values of the identified connector hubs and BDI-II scores in patients. Significant correlations were observed between BDI-II scores and the FCOR values of L PoG with SMN (r = 0.1472, p = 0.027), L PrG with Lang (r = 0.1440, p = 0.031), R SMA with Visu (r = 0.1309, p = 0.05), R PrG with Aud (r = 0.1430, p = 0.032), hVis (r = 0.1407, p = 0.035), Prec (r = 0.1351, p = 0.043), pVis (r = 0.1527, p = 0.022), and Visu (r = 0.1416, p = 0.034), L VLN with pSal (r = 0.1377, p = 0.039) and Prec (r = 0.1521, p = 0.022), R VLN with Prec (r = 0.2445, p < 0.001), R MCgG with BG (r = 0.1406, p = 0.035), R Pu with Prec (r = 0.1394, p = 0.037), and R PHG with SMN (r = -0.1348, p = 0.043).

## 4 Discussion

Using FCOR, we have identified regions with altered connections across several large-scale functional networks in patients with MDD compared to controls. The connections with BG exhibited the most widespread alterations, followed by that of SMN. Among affected regions, we have identified connector hubs located in the sensorimotor cortex, thalamus, cerebellum, and limbic regions with connections across multiple RSNs that were significantly altered in MDD. Sensorimotor connector hubs had significantly lower connections with primary processing, default mode (vDMN, Prec), and visuospatial networks, but more widespread connections with anterior salience. On the other hand, cerebellar and thalamic connector hubs exhibited altered connections with the core neurocognitive, BG, and language networks, whereas limbic connector hubs had altered connections with salience and BG networks. Additionally, some of these altered connections correlated with depression scores in patients. These findings suggest that MDD is characterized not only with connectivity alterations within the fronto-limbic system as previously considered, but by a more widespread impairment of the brain’s integrative functions, potentially driven by the altered connectivity of the identified connector hubs and the BG network.

One of our main findings is the impact of MDD on the connectivity of sensorimotor connector hubs with the primary processing (sensory, motor, visual, and auditory), default mode and visuospatial networks, potentially reflecting a breakdown in somatosensory integration and top-down motor control. In healthy participants, primary processing networks are closely linked together (Bagarinao et al., 2020), which was significantly reduced in the patient group. Sensorimotor connector hub regions play a critical role in the integration of sensory-motor information, not only among primary processing systems, but also with the visuospatial network (dorsal attention network), responsible for top-down control of visuospatial attention (Corbetta and Shulman, 2002). Their impairment suggests that the integration of sensory-motor information within the primary processing systems are significantly impaired in MDD, consistent with some of the observed clinical manifestations of this disorder such as psychomotor retardation (e.g., slow speaking rate, delayed motor initiation, reduced speed, and body immobility (Bennabi et al., 2013)), reduced visual contrast sensitivity (Bubl et al., 2010), and impaired auditory processing of nonspeech stimuli (Zwanzger et al., 2012), among others. Other reported connectivity alterations in the primary processing systems in MDD (Gallo et al., 2023, Javaheripour et al., 2021, Yang et al., 2021, Zarrinpar et al., 2006) may also be associated with the impairment of these connector hubs. Interestingly, similar impairment of sensorimotor connector hub regions was also reported in Parkinson’s disease (PD) with the degree of FCOR alterations correlating with the patients’ motor symptoms (Bagarinao et al., 2022). In PD, bradykinesia is one of the cardinal symptoms, which can be considered an extreme example of psychomotor retardation.

Connectivity alterations of cerebellar and thalamic connector hubs were also observed in MDD. The existence of connector hubs in the cerebellum and thalamus in healthy controls had been demonstrated in previous studies (Greene et al., 2020, Kawabata et al., 2022, Kawabata et al., 2021). Cerebellar connector hub regions were localized in the posterior cerebellum, especially in lobules VI, VII, and IX and were mainly connected with the core neurocognitive networks (Kawabata et al., 2022). In the thalamus, prominent connector hubs included the anteroventral, ventral lateral, and mediodorsal nuclei with functional connections across multiple networks (Kawabata et al., 2021). Damage to these connector hubs could result in impaired integration across multiple domains. Thalamic hub dysfunction, in particular, is associated with widespread deficits in executive, language, and memory functions, as revealed by neuropsychological studies of patients with focal thalamic lesions (Hwang et al., 2021). Our findings have shown that some of these connector hubs, specifically those in the bilateral posterior cerebellum and the ventral lateral nucleus of the thalamus, are indeed impaired in MDD with lower connectivity to both primary processing (less anti-correlation) and cognitive networks (less correlation). Other evidence implicating the cerebellum in MDD included reduced connectivity with the executive control networks, particularly to the dorsolateral prefrontal cortex, and the default mode networks in patients with depression (Alalade et al., 2011, Liu et al., 2012, Zhu et al., 2020), consistent with our findings. In the thalamus, hyperconnectivity was observed in patients compared to controls (Brown et al., 2017, Greicius et al., 2007, Hamilton et al., 2015), characterized treatment-resistant depression (Yamamura et al., 2016), and even served as a prominent signature for the classification of MDD (Gallo et al., 2023). Connectivity alterations of cerebellar and thalamic connector hubs were similarly observed in patients with schizophrenia (Yamamoto et al., 2022). Given the shared symptoms observed in these disorders, these connector hub alterations may underly the common neurological background between these disorders.

Impaired connector hubs were also identified in limbic regions including those in the cingulate gyrus (R dACC, L MCgG, R PCC) and parahippocampal gyrus (R PHG). The cingulate cortex subserved several functions including cognitive, emotional, motor, nociceptive, and visuospatial (Vogt et al., 1992). The anterior cingulate cortex, in particular, can be subdivided into an affective and a cognitive component with the former involved in the assessment of motivational content and assigning emotional valence to internal and external stimuli, whereas the latter engaged in both response selection and cognitively demanding information processing (Bush et al., 2000, Devinsky et al., 1995). Thus, the anterior cingulate cortex provides a mechanism by which affect, sensory, motor, and cognition can be integrated. The identified connector hub region in the dorsal anterior cingulate cortex could facilitate the integration of these processes. Given the involvement of the dorsal anterior cingulate cortex in response selection, error detection, and conflict monitoring, among others, its functional disruption in MDD may contribute to impaired initiation and organization of behavior (Devinsky et al., 1995) and its dysconnectivity with the salience network may lead to the observed emotional dysregulation from executive control. The posterior cingulate cortex, on the other hand, is involved in autobiographical memory and self-referential processes (Buckner and Carroll, 2007) and a key component of the default mode network. Thus, the observed increased in connectivity between the connector hub in the right posterior cingulate cortex and executive control network may suggest preference to content of internal thoughts, especially considering the increased connections of the right parahippocampal gyrus with the default mode network. Furthermore, the observed increased connectivity of the posterior cingulate cortex with the medial prefrontal cortex also supports rumination (Hamilton et al., 2015), a common symptom of MDD.

The network with the most altered connectivity in MDD is the basal ganglia. Although it has been classically associated with motor and movement disorders, several studies have now shown basal ganglia’s involvement in several psychiatric disorders. The basal ganglia is traversed by several parallel cortico-subcortical circuits (Alexander et al., 1986) that act to control motor, cognitive, and affective functions. Alterations of these circuits could lead to the various psychiatric disorders including MDD, schizophrenia, and others (Macpherson and Hikida, 2019). For instance, MDD’s effect on the motor/oculomotor circuitry may lead to psychomotor symptoms. Alterations in orbitofrontal/anterior cingulate circuitry may also manifest in the persistent low mood and low motivation as well as the occasional delusions and hallucinations typically observed in MDD. Consistent with this, our results showed that most of the identified connector hubs have altered connections with the BG network, potentially involving different basal ganglia circuitries. Our recent study on schizophrenia (Yamamoto et al., 2022) also showed similar widespread connectivity alterations in BG network, suggesting similarities between these disorders.

Overall, the dysconnectivity of the connector hubs located in the sensorimotor cortices suggests that integration of the primary processing systems is compromised in MDD. Information flow between primary processing and the core neurocognitive networks may also be impaired with the dysconnectivity of the cerebellar and thalamic connector hubs with the core neurocognitive networks coupled with the dysconnectivity of the visuospatial network with the sensorimotor connector hubs. The dysconnectivity of the dorsal anterior cingulate cortex, an important component of the salience network, could also impair the integration of sensorimotor, cognitive, and affective processes in the brain. Together, this lack of integration of external sensory information at different processing levels in the brain may bias the focus toward internal and ruminative thinking, as evidenced by the strong connections of the PCC connector hub with the executive control network and the parahippocampal connector hub with the default mode network.

In terms of methodology, our use of the FCOR metric as compared to classical approaches that used specific node-to-node or network-to-network analysis offered several advantages and has provided additional insights beyond conventional methods. FCOR method directly quantifies the degree to which key connector hubs participate across multiple networks, providing a more global and integrative measure of network dysfunction. FCOR also allows the simultaneous detection of both hypo- and hyperconnectivity patterns, like the increased salience network connectivity in the context of widespread hypoconnectivity elsewhere, catching subtler compensatory or maladaptive changes. With FCOR, network intermediaries can also be identified such as the one observed with sensorimotor connector hubs, indicating a shift away from adaptive information integration and toward over-salience of distressing internal signals. Another point to consider is the influence of head motion, which could affect functional connectivity estimation using resting-state functional MRI (Power et al., 2015). From Table 1, only about 27% of the participants have mean frame-wise displacement values that were greater than 0.2 mm, indicating that most participants had relatively minimal head motion. Additionally, no significant difference in mean frame-wise displacement was observed between patients and controls from all sites, potentially balancing out head motion effects in the comparisons between groups. While this may not fully eliminate the effects of head motion, this does minimize them.

Finally, we would like to mention some limitations of this study. First, due to the limited clinical data made available with the shared MRI data, we were only able to examine the association between the FCOR values of affected hub regions and BDI-II scores. The relationship between FCOR values of affected connector hub regions with the other clinical characteristics of the patient group needs to be characterized in future studies. Second, we were also unable to investigate the effect of medication on our results given the limited information regarding medication in this cohort. Altered connectivity of the core neurocognitive networks may be influenced by medication but that of SMN could be robust (Javaheripour et al., 2021). Third, FCOR values depend on the choice of reference RSNs, and so the identified connector hubs are relative to the chosen templates. Additionally, the operational definition of connector hubs is not standardized, and different hub metrics or thresholding rules may yield divergent results. However, our prior work demonstrates that FCOR is relatively robust for identifying connector hubs when using alternative RSN templates, both in whole-brain analyses and between select RSNs such as between control and primary processing networks or control and default mode networks (Bagarinao et al., 2020). Fourth, correction for multiple comparisons was mainly done within RSNs for whole-brain FCOR analyses and within ROIs for seed-based connectivity analyses. Applying global corrections across RSNs or seed ROIs would make the identification of connectivity changes robust. Lastly, there were also differences in the duration of the resting-state functional MRI scans across sites with 3 of the 6 sites having only about 4.5 min of data. Although previous studies have shown that the strength of functional connectivity tended to stabilize at around 5 min of data (Braun et al., 2012, Van Dijk et al., 2010), the reliability of the estimates have been shown to significantly increase with longer scan times (Birn et al., 2013). Given this, the shorter scan times in some data could affect the stability of the constructed connectivity maps from which the FCOR values were estimated. Thus, our results need to be interpreted under these limitations.

## 5 Conclusion

Using FCOR, we have identified connector hub regions with connections across several large-scale functional networks that were significantly altered in MDD. Hypoconnectivity of sensorimotor, thalamic, cerebellar, and limbic connector hubs with primary processing and neurocognitive networks together with selective hyperconnectivity of default mode connector hubs reflect an imbalance between externally oriented and introspective/self-referential processing. This disrupted connectivity at key hub regions could undermine the brain’s ability to exert top-down control over emotional, cognitive, and sensory inputs. These findings suggest that MDD is characterized not only with connectivity alterations within the fronto-limbic system but also by the more widespread impairment of the brain’s integrative functions involving sensory, motor, cognitive, and affective processes, which may help explain the varying and heterogeneous symptoms observed in this disorder.

## Funding sources

This research did not receive any specific grant from funding agencies in the public, commercial, or not-for-profit sectors

## CRediT authorship contribution statement

**Norika Hayashi:** Writing – original draft, Investigation, Formal analysis. **Epifanio Bagarinao:** Writing – review & editing, Validation, Supervision, Software, Resources, Methodology, Formal analysis, Conceptualization.

## Declaration of competing interest

The authors declare that they have no known competing financial interests or personal relationships that could have appeared to influence the work reported in this paper.

## Data Availability

This study used publicly available MRI data from the Strategic Research Program for the Promotion of Brain Sciences (SRPBS) Multi-Disorder MRI Dataset

## Appendix A Supplementary data

Supplementary Data 1 The supplementary material contains detailed imaging parameters of the MRI data used in this study, additional figures showing distribution of participants in terms of age, sex, and mean frame-wise displacement, contrast maps for the FCOR analysis and seed-based connectivity analysis, and a table listing regions showing significant changes in FCOR values in patients compared to controls.
