# Supplementary material for: Failure to integrate: Connector hub dysfunction in major depressive disorder
Source: Neuroimage Clin. 2026 May 19;50:104010. doi: 10.1016/j.nicl.2026.104010 (PMC13224140; doi:10.1016/j.nicl.2026.104010)
Supplement: Supplementary Data 1 — The supplementary material contains detailed imaging parameters of the MRI data used in this study, additional figures showing distribution of participants in terms of age, sex, and mean frame-wise displacement, contrast maps for the FCOR analysis and seed-based connectivity analysis, and a table listing regions showing significant changes in FCOR values in patients compared to controls. [file mmc1.pdf]

Failure to Integrate: Connector hub dysfunction in major depressive disorder

Norika Hayashi [a], Epifanio Bagarinao [b,c]

[a] Department of Nursing, School of Health Sciences, Faculty of Medicine, Nagoya University, Nagoya, Aichi, Japan

[b] Brain and Mind Research Center, Nagoya University, Nagoya, Aichi, Japan

[c] Department of Integrated Health Sciences, Nagoya University Graduate School of Medicine, Nagoya, Aichi, Japan

Supplementary Materials

Imaging Parameters

Hiroshima University Hospital (HUH)

MRI data were acquired using a GE Signa HDxt. T1-weighted (T1w) images were acquired with the following imaging parameters: voxel size 1 × 1 × 1 mm^3, repetition time (TR) = 6812 ms, echo time (TE) = 1896 ms, inversion time (TI) = 450 ms, flip angle (FA) = 20 degrees, field of view (FOV) = 256 mm, and 256 × 256 matrix dimension. For the resting-state functional MRI data, the following imaging parameters were used: TR = 2s, TE = 27ms, FA = 90 degrees, FOV = 256mm, 64×64 matrix dimension, 143+7 (dummy) slices, and total scan time of 4 min 46 sec + 14 sec (dummy).

Hiroshima Rehabilitation Center (HRC)

MRI data from Hiroshima Rehabilitation Center were acquired using a GE Signa HDxt MRI scanner. T1w images used the following imaging parameters: voxel size 1 × 1 × 1 mm^3, TR = 6812 ms, TE = 1896 ms, TI = 450 ms, FA = 20°, FOV = 256 mm, 256 × 256 matrix dimensions. Resting-state fMRI images were acquired with the following imaging parameters: TR = 2s, TE = 27ms, FA = 90°, FOV = 256mm, matrix dimensions 64 × 64, 143+7 (dummy) slices, and total scan time of 4 min 46 sec + 14 sec (dummy).

Hiroshima Kajikawa Hospital (HKH)

Hiroshima Kajikawa Hospital used a SIEMENS Spectra MRI scanner for data acquisition. T1w images were acquired with the following imaging parameters: voxel size 1 × 1 × 1 mm^3, TR = 1900 ms, TE = 2.38 ms, TI = 900 ms, FA = 10°, FOV = 256 mm, 256 × 256 matrix dimensions. Resting-state functional MRI images were acquired with the following imaging parameters: TR = 2.7s, TE = 31ms, FA = 90°, FOV = 256mm, 64 x 64 matrix dimensions, 107+5 (dummy) slices, and total scan time 4 min 49 sec + 14 sec (dummy).

Center of Innovation (COI) in Hiroshima University

The Center of Innovation in Hiroshima University has a SIEMENS MAGNETOM Verio Dot MRI scanner. T1w images were acquired with the following imaging parameters: voxel size = 1 × 1 × 1 mm^3, TR = 2300

ms, TE = 2.98 ms, TI = 900 ms, FA = 900 ms. The resting-state functional MRI images were acquired with the following imaging parameters: TR = 2300 ms, TE = 2.98 ms, TI = 900 ms, FA = 9°, FOV = 256 mm, 256 × 256 matrix dimensions. TR = 2.5s, TE = 30ms, FA = 80°, FOV = 212mm, 64x64 matrix dimensions, 240+4 (dummy) slices, total scan time 10 minutes + 10 seconds (dummy).

Kyoto University (KUT)

Kyoto University used a SIEMENS TimTrio MRI scanner. T1w images were acquired with the following imaging parameters: voxel size = 0.9375 x 0.9375 x 1 mm^3, TR = 2000 ms, TE = 3.4 ms, TI = 990 ms, FA = 8°, FOV = 225 × 240 mm, 240 × 256 matrix dimensions. The resting-state functional MRI images were acquired with the following imaging parameters: TR = 2.5s, TE = 30ms, FA = 80°, FOV = 212 × 212mm, 64 × 64 matrix dimensions, 240+4 (dummy) slices, total scan time of 10 min + 10 sec (dummy).

Tokyo University (UTO)

MRI data from Tokyo University were acquired using a GE Discovery MR750w MRI scanner. T1w images were acquired with the following imaging parameters: voxel size = 1 × 1 × 1.2 mm3, TR = 7.7 ms, TE = 3.1 ms, TI = 400 ms, FA = 11°, FOV = 240 mm, 256 × 256 matrix dimensions. Resting-state functional MRI images were acquired with the following imaging parameters: TR = 2.5s, TE = 30ms, FA = 80°, FOV = 212mm, 64x64 matrix dimensions, 240+4 (dummy) slices, total scan time 10 minutes + 10 seconds (dummy).

Supplementary Figures

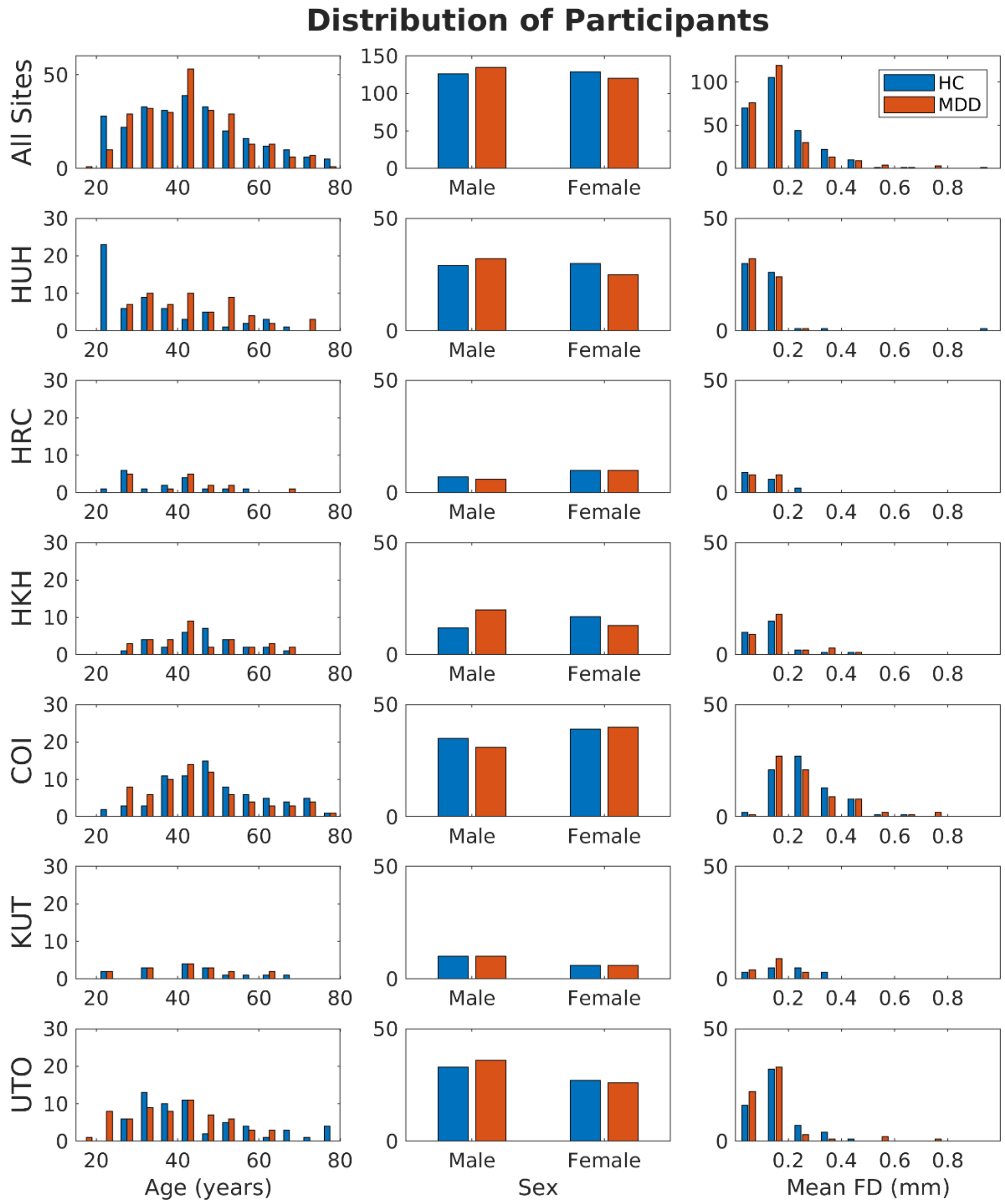

Supplementary Figure S1. Distribution of age (left column), sex (middle column), and mean framewise displacement (FD) values (right column) of all participants included in the analysis as well as for each site. HC, healthy controls; MDD, patients with major depressive disorder.

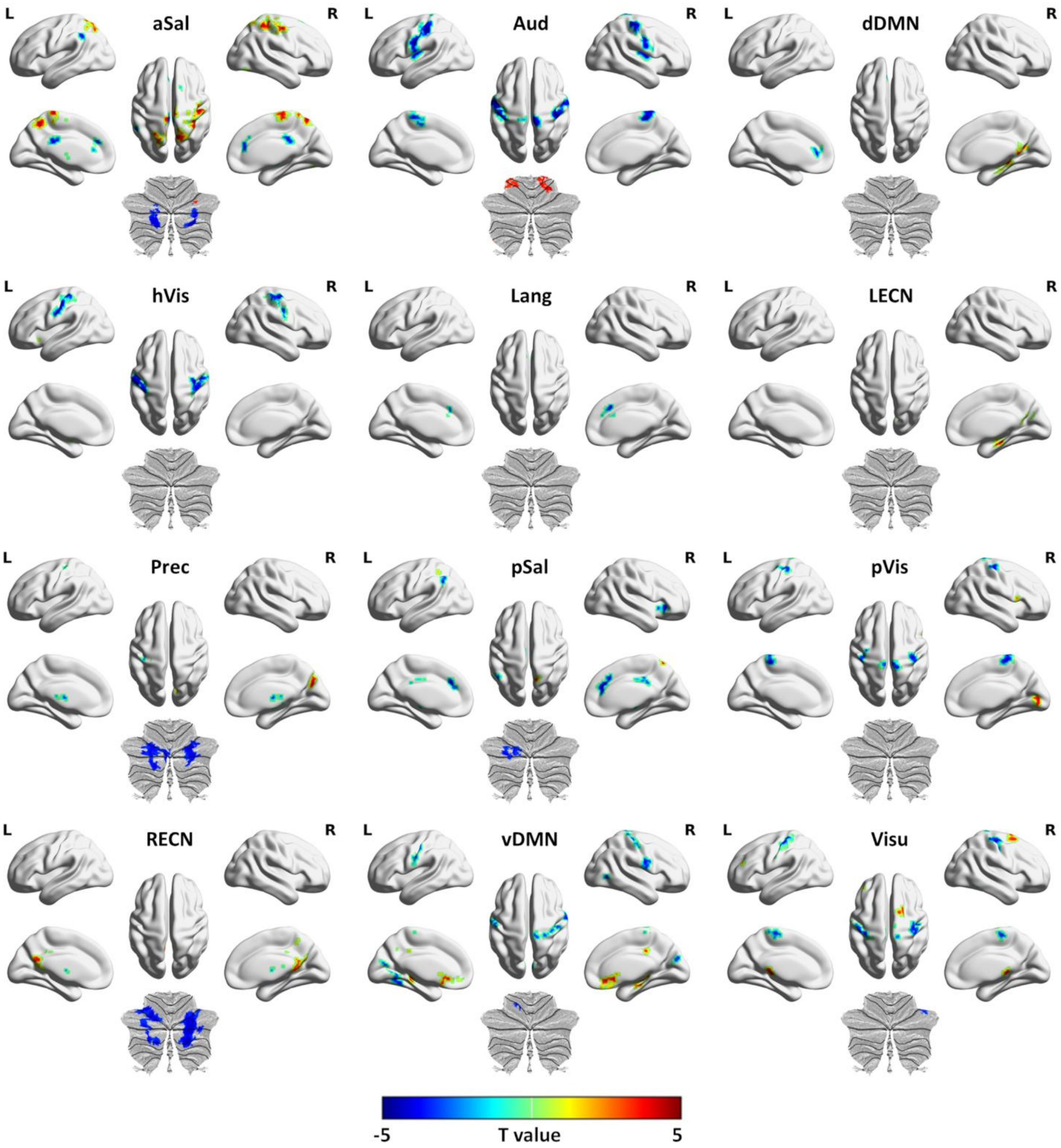

Supplementary Figure S2. Contrast maps showing regions with significant alterations in FCOR values in the patient group compared with the control group. Regions with lower FCOR values in patients are shown in blue, whereas those with higher FCOR values are shown in red. The list of clusters including the peak's MNI coordinates and cluster size is given in Supplementary Table S1.

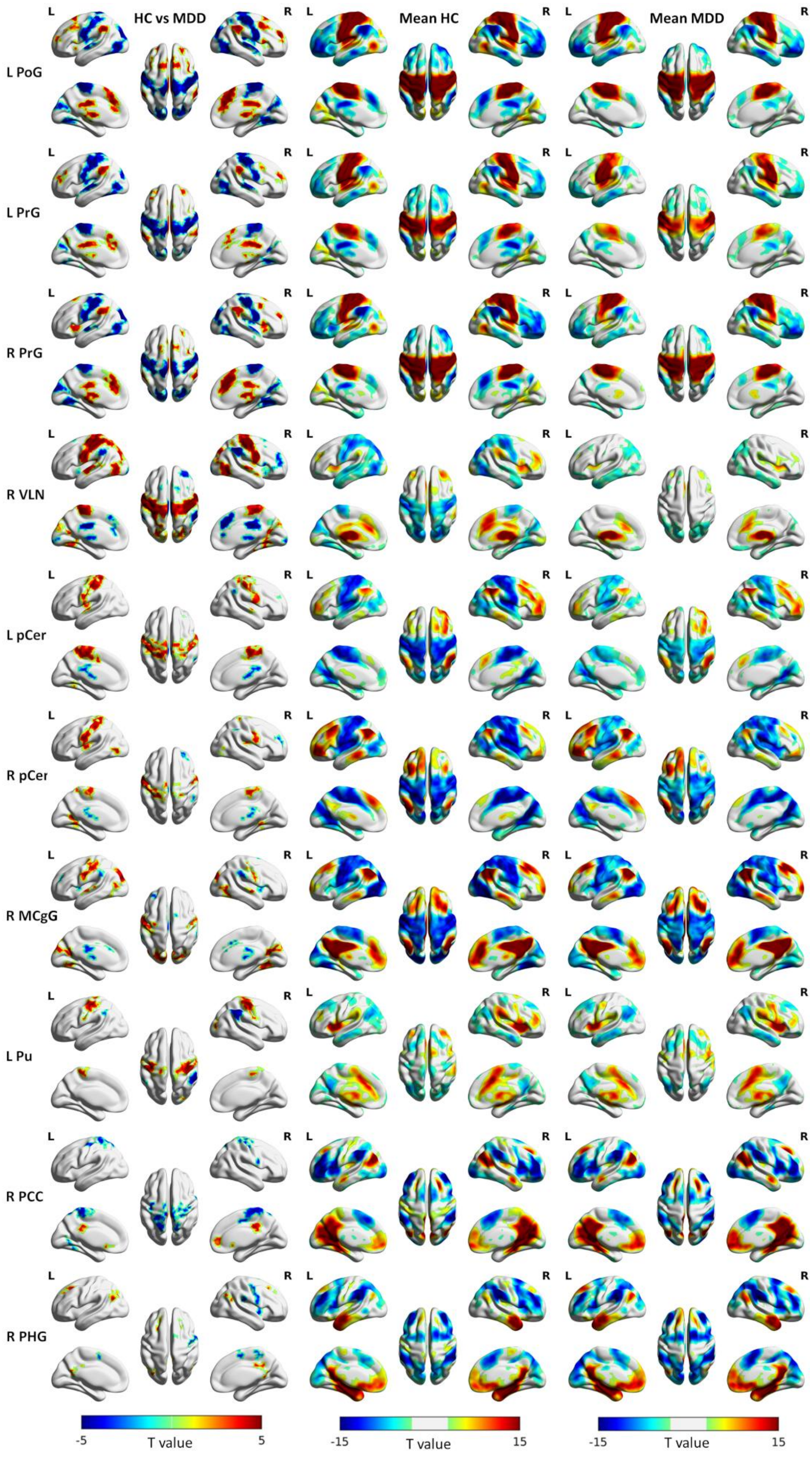

HC vs MDD
Mean HC
Mean MDD
L
R
L PoG
L PrG
R PrG
R VLN
L pCer
R pCer
R MCgG
L Pu
R PCC
R PHG
-5
T value
5
-15
T value
15
-15
T value
15

Supplementary Figure S3. Seed-based connectivity analysis of identified connector hubs. Left column figures are contrast maps showing regions exhibiting significant difference in functional connectivity between patients with major depressive disorder (MDD) and healthy controls (HC) for the identified connector hubs not shown in Figure 4. Blue colors indicate lower connectivity in the patient group, whereas red colors indicate higher connectivity. The mean functional maps for the control group are shown in the middle column (Mean HC), whereas those of the patient group are shown in the rightmost column (Mean MDD). For the mean connectivity maps, red colors indicate positive connectivity values (positive correlation), whereas blue colors indicate negative connectivity values (anti-correlation). From top to bottom: L PoG, left postcentral gyrus; L PrG, left precentral gyrus; R PrG, right precentral gyrus; R VLN, right ventrolateral nucleus of the thalamus; L pCer, left posterior cerebellum; R pCer, right posterior cerebellum; R MCgG, right midcingulate gyrus; L Pu, left putamen; R PCC, right posterior cingulate cortex; R PHG, right parahippocampal gyrus.

Supplementary Table

Supplementary Table S1. Regions showing significant alterations in FCOR values in patients with major depressive disorder (MDD) compared to healthy controls (HC).

RSN	Contrast	X	Y	Z	z-Value	Cluster Size	Peak Location	Other Peaks
**aSal**	HC > MDD	18	-15	12	5.77	193	R ThP	
		-12	-15	12	5.7	125	L ThP	
		30	-72	-42	5.49	52	R Cer	
		-30	-78	-42	4.91	87	L Cer	
		-60	-51	48	4.73	55	L SMG	
		27	6	72	4.66	38	R SFG	
		-6	-27	27	4.57	81	L MCgG	R PCgG
		-3	33	12	4.37	89	L ACgG	R ACgG
	HC < MDD	42	-21	51	4.97	273	R PoG	R SPL
		-12	-54	51	4.9	182	L PCu	L SPL
		12	-60	60	4.71	109	R SPL	R PCu
		30	-72	-6	4.59	44	R OFuG	
		-6	-30	72	4.58	133	L PrG	R MPrG
**Aud**	HC > MDD	-33	-9	12	5.97	52	L AIns	L CO
		39	-24	57	5.49	288	R PoG	
		-45	-27	51	5.43	358	L PoG	L PrG
		33	-9	12	5.33	64	R PIns	R AIns
		9	-30	66	4.88	220	R MPrG	L MPrG, R PoG
	HC < MDD	30	-45	-24	4.32	51	R Cer	
		-24	-45	-21	4.02	36	L Cer	
**BG**	HC > MDD	-3	-24	27	6.04	196	L MCgG	R PCgG
		48	12	57	5.66	63	R MFG	
		-30	-78	-45	5.28	327	L Cer	
		-57	-51	36	4.92	138	L SMG	L AnG
		15	-78	-30	4.91	210	R Cer	
		18	-27	9	4.83	30	R ThP	
		-15	-15	12	4.76	91	L ThP	
		18	21	66	4.61	95	R SFG	
		60	-45	45	4.54	249	R SMG	
		27	57	30	4.43	106	R SFG	R MFG
		3	27	45	4.3	59	R MSFG	
	HC < MDD	-36	-21	54	5.53	1382	L PrG	L PoG, R PrG
		-3	-6	15	4.78	41	L ThP	R Cau, L Cau
		-18	-63	51	4.77	39	L SPL	
**dDMN**	HC > MDD	15	-9	9	4.8	31	R ThP	
		-6	39	9	4.62	34	L ACgG	L MSFG
	HC < MDD	15	-60	18	4.48	70	R PCu	R PCgG
		27	-36	-6	4.4	52	R Hip	

**hVis**	HC > MDD	39	-15	57	4.96	267	R PrG	R PoG
		-39	-30	57	4.88	230	L PoG	
	HC < MDD	-24	0	-6	4.79	84	L Pu	
**Lang**	HC > MDD	6	24	39	4.08	93	R SMC	L ACgG, L MSFG
**LECN**	HC > MDD	21	-18	21	4.42	46	R ThP	R Cau
		-12	-3	9	4.15	76	L ThP	L Pu, L Cau
	HC < MDD	24	-21	-18	5.61	48	R Hip	
		9	-63	18	4.59	38	R Cun	
**Prec**	HC > MDD	12	-9	6	5.54	239	R ThP	L ThP
		24	-69	-30	5.23	108	R Cer	
		6	-18	-15	4.76	83	Brainstem	
		-27	-75	-39	4.41	114	L Cer	
		-33	-21	72	4.09	39	L PrG	L PoG
	HC < MDD	12	-72	27	4.73	43	R Cun	
**pSal**	HC > MDD	12	-6	6	5.84	254	R ThP	
		-6	30	30	5.19	152	L ACgG	R MSFG, R SMC
		-9	-9	0	5.09	164	L ThP	L Cau
		36	24	-3	4.53	64	R AIns	R Pu
		-48	-51	36	4.53	53	L SMG	L AnG
		-21	-69	-27	3.96	33	L Cer	
		3	-18	36	3.87	35	R MCgG	R PCgG, L PCgG
	HC < MDD	15	-57	60	4.93	50	R SPL	
		-27	-45	42	4.19	50	L SPL	
**pVis**	HC > MDD	-9	-36	69	4.82	175	L PoG	R PoG, R MPrG
		36	-21	57	4.31	93	R PrG	R PoG
		-36	-12	60	4.14	63	L PrG	L PoG
	HC < MDD	12	-78	3	4.58	65	R Calc	
		60	15	3	4.57	31	R OpIFG	R CO
**RECN**	HC > MDD	15	-3	9	6.05	586	R ThP	L ThP
		21	-72	-39	5.34	250	R Cer	
		-27	-63	-24	4.79	116	L Cer	
	HC < MDD	9	-63	18	4.8	215	R Cun	R PCgG
		-3	-45	36	3.97	40	L PCgG	R PCu
**SMN**	HC > MDD	9	-36	75	5.78	247	R PoG	L MPrG, L PrG
		-45	-24	48	5.69	366	L PoG	L CO
		63	-3	21	5.56	266	R PoG	R CO, R PrG
		-18	-81	27	5.31	603	L SOG	L Cun
		-57	-24	3	4.64	100	L STG	
		21	-72	-6	4.55	71	R LiG	
		-24	-69	-9	4.41	31	L OFuG	L LiG
		60	-6	0	4.31	113	R STG	R TMP
	HC < MDD	54	15	3	5.39	182	R OpIFG	R FO
		39	3	48	5.06	52	R MFG	R PrG
		-45	6	3	4.83	55	L CO	L FO
		6	-57	-18	4.69	201	R Cer	L Cer
		21	-30	-33	4.51	39	R Cer	

		-24	0	-6	4.42	30	L Pu	
		0	-36	3	4.31	51	Brainstem	R PCgG
		-30	12	15	4.23	39	L AIns	L FO
		0	-9	24	4.13	32	L MCgG	R MCgG
		0	6	54	4.03	53	L SMC	R SMC
		60	-30	42	3.95	29	R SMG	
		63	-27	24	3.84	34	R PO	R SMG
		30	45	24	3.79	29	R MFG	R SFG
**vDMN**	HC > MDD	45	-63	3	4.6	30	R MTG	R IOG
		36	-18	48	4.47	172	R PrG	R SPL
		54	-6	21	4.42	71	R PoG	R PrG
		0	-72	15	4.42	80	L Cun	L Calc, R Cun
		-21	-57	-12	4.08	59	L LiG	L FuG
		-54	-15	42	3.68	33	L PoG	L PrG
	HC < MDD	3	-39	42	4.93	63	R PCgG	
		27	-21	-15	4.73	57	R Hip	
		0	18	-9	4.44	135	L SCA	R ACgG
		-27	-33	-9	4.13	33	L Hip	
		0	-66	45	4.05	30	L PCu	
**Visu**	HC > MDD	33	-18	45	5.34	197	R PrG	R PoG
		-33	-24	45	4.62	240	L PrG	L PoG
		18	-33	78	4.12	46	R PoG	R PrG
		-3	-27	66	4.09	65	L MPrG	R MPrG
		54	-54	-18	4.06	40	R ITG	R FuG
	HC < MDD	-12	-30	3	4.51	95	L ThP	R ThP
		-42	45	24	4.45	41	L MFG	
		21	6	57	4.1	37	R SFG	

Abbreviations: ACgG, anterior cingulate gyrus; AIns, anterior insula; AnG, angular gyrus; aSal, anterior salience network; Aud, auditory network; BG, basal ganglia network; Calc, calcarine; Cau, caudate; Cer, cerebellum; CO, central operculum; Cun, cuneus; dDMN, dorsal default mode network; FCOR, functional connectivity overlap ratio; FO, frontal operculum; FuG, fusiform gyrus; HC, healthy controls; Hip, hippocampus; hVis, high visual network; IOG, inferior occipital gyrus; ITG, inferior temporal gyrus; L, left; Lang, language network; LECN, left executive control network; LiG, lingual gyrus; MCgG, midcingulate gyrus; MDD, patients with major depressive disorder; MFG, middle frontal gyrus; MPrG, medial precentral gyrus; MSFG, medial segment of the superior frontal gyrus; MTG, middle temporal gyrus; OFuG, occipital fusiform gyrus; OpIFG, opercular part of the inferior frontal gyrus; PCgG, posterior cingulate gyrus; PCu, precuneus; PIns, posterior insula; PO, posterior operculum; PoG, postcentral gyrus; Prec, precuneus network; PrG, precentral gyrus; pSal, posterior salience network; Pu, putamen; pVis, primary visual network; R, right; RECN, right executive control network; RSN, resting state networks; SCA, subcallosal area; SFG, superior frontal gyrus; SMC, supplementary motor cortex; SMG, supramarginal gyrus; SMN, sensorimotor network; SOG, superior occipital gyrus; SPL, superior parietal lobule; STG, superior temporal gyrus; ThP, thalamus proper; TMP, temporal pole; vDMN, ventral default mode network; Visu, visuospatial network;
